# Using gamma index to flag changes in anatomy during image‐guided radiation therapy of head and neck cancer

**DOI:** 10.1002/acm2.12180

**Published:** 2017-09-13

**Authors:** Bryan Schaly, Jeff Kempe, Varagur Venkatesan, Sylvia Mitchell, Jerry J Battista

**Affiliations:** ^1^ Departments of Oncology and Medical Biophysics Western University London ON Canada; ^2^ Physics and Engineering London Regional Cancer Program London ON Canada; ^3^ Schulich School of Medicine and Dentistry Western University London ON Canada; ^4^ Department of Radiation Oncology London Regional Cancer Program London ON Canada; ^5^ Department of Radiation Therapy London Regional Cancer Program London ON Canada

**Keywords:** adaptive radiation therapy, anatomical variations, gamma index, head and neck cancer, image‐guided radiation therapy

## Abstract

During radiation therapy of head and neck cancer, the decision to consider replanning a treatment because of anatomical changes has significant resource implications. We developed an algorithm that compares cone‐beam computed tomography (CBCT) image pairs and provides an automatic alert as to when remedial action may be required. Retrospective CBCT data from ten head and neck cancer patients that were replanned during their treatment was used to train the algorithm on when to recommend a repeat CT simulation (re‐CT). An additional 20 patients (replanned and not replanned) were used to validate the predictive power of the algorithm. CBCT images were compared in 3D using the gamma index, combining Hounsfield Unit (HU) difference with distance‐to‐agreement (DTA), where the CBCT study acquired on the first fraction is used as the reference. We defined the match quality parameter (MQP_x_) as a difference between the *x* ^th^ percentiles of the failed‐pixel histograms calculated from the reference gamma comparison and subsequent comparisons, where the reference gamma comparison is taken from the first two CBCT images acquired during treatment. The decision to consider re‐CT was based on three consecutive MQP values being less than or equal to a threshold value, such that re‐CT recommendations were within ±3 fractions of the actual re‐CT order date for the training cases. Receiver‐operator characteristic analysis showed that the best trade‐off in sensitivity and specificity was achieved using gamma criteria of 3 mm DTA and 30 HU difference, and the 80^th^ percentile of the failed‐pixel histogram. A sensitivity of 82% and 100% was achieved in the training and validation cases, respectively, with a false positive rate of ~30%. We have demonstrated that gamma analysis of CBCT‐acquired anatomy can be used to flag patients for possible replanning in a manner consistent with local clinical practice guidelines.

## 1 INTRODUCTION

Radiation therapy of head and neck cancer is complex especially if the gross disease and possible nodal regions at risk are located in close proximity to several critical structures. Precision radiation therapy increases the probability of success and reduces the risk and severity of complications. Volumetric modulated arc therapy (VMAT) produces dose distributions with steep gradients in order to minimize dose to neighboring healthy organs. Therefore, daily image‐guided radiation therapy (IGRT) is necessary to ensure accurate target localization during treatment. In addition, it is common for some patients to experience tumor regression or weight loss during treatment, which may result in anatomical changes that can affect dose delivery to the tumor and organs at risk.^1^ Such changes may require the patient to have a repeat CT (re‐CT) simulation with the possibility of generating a revised treatment plan based on the changes in anatomy.^2^, ^3^, ^4^, ^5^ At our institution, radiation therapists are responsible for documenting changes in patient anatomy as treatment progresses. If anatomical changes are judged to be substantial, the physicist and/or the clinical specialist in radiation therapy (CSRT) is called to make a recommendation to the radiation oncologist as to whether a re‐CT simulation is required. This recommendation is based on assessing the potential overdosing of critical organs such as spinal cord and/or if there are visual changes of the gross disease which could result in suboptimal dose coverage to the high dose target volume. Before a decision to re‐CT is made, the physicist and CSRT (and perhaps the physician) review several image matches offline to look at systematic trends and assess the magnitude of volume changes. This approach can be time consuming and is dependent on the judgment of several observers. Therefore, we have developed a method to automatically compare cone‐beam CT (CBCT) images offline mathematically and provide an alert to the physician when action may be required. The alert provided by the algorithm is based on decision thresholds that are derived from retrospective analysis of CBCT image comparisons combined with re‐CT decisions that were made on actual patient cases. Therefore, the algorithm is *trained* to flag changes in anatomy in a manner consistent with local practice. The overall goal is to improve the efficiency in the decision‐making process, since many patients that are reviewed for changes in anatomy do not result in re‐CT recommendations. A secondary goal is to provide a quality assurance safeguard to human judgment of anatomical changes.

The question of when to replan during head and cancer treatment has been studied by other investigators. Paganelli et al^6^ used deformable image registration (DIR) to quantify how much tissue deformation had occurred after 40–50 Gy dose delivery. Stoiber et al^7^ used DIR to calculate a couch shift from a displacement vector field. Replanning was considered if the resulting couch shift did not result in the targets and critical structures being positioned within some chosen margin. Lai et al^8^ correlated thickness and circumference at different levels of the neck to IGRT displacement. They determined that replanning should be considered for patients who have had large decreases in thickness and circumference at the level of the mastoid tip. Our goal is to develop a method based on image comparison and generate decision criteria based on our clinical process. This is more generic since there are no specific dose thresholds and there is no need to outline structures on CBCT images.

We propose to use the gamma index^9^ to compare CBCT image data for the purpose of monitoring changes in anatomy during treatment. There are other metrics that can be used to evaluate image registration quality or similarity. Wu and Murphy^10^ developed a neural network approach to determine whether a 3D/3D bony registration was successful or unsuccessful as applied to head and cancer radiation therapy. They compared two metrics: mutual information and mean‐squared intensity difference. Castadot et al^11^ compared 12 DIR algorithms as applied to adaptive radiation therapy of head and neck cancer using the Dice similarity index^12^ and the correlation coefficient. We chose the gamma index because of our familiarity with the method (through dose comparison) and it has the feature of defining pass/fail criteria for all pixels in the 3‐D image and a pass/fail map can be generated (i.e., the gamma map).

The study design is carried out in two parts: (a) algorithm training and (b) validation of the alert system. For algorithm training, we use retrospective CBCT data from ten patients that were replanned and calculate gamma maps from CBCT comparisons. The gamma maps are based on CBCT number differences and distance‐to‐agreement between two imaging data sets. From this, we define a match quality parameter (MQP) that tracks the level of anatomy mismatch, such that plotting this parameter by fraction number shows a downward trend as the degree of mismatch worsens. Then, the downward trend pattern is compared to when re‐CT simulation was actually ordered by the radiation oncologist to determine the alert signal decision threshold to recommend a re‐CT. The optimum parameter set is chosen based on receiver‐operator characteristic (ROC) analysis^13^ that assesses the sensitivity and specificity of the alert software. For algorithm validation, we test the algorithm on twenty different patients: ten patients that were replanned and ten patients that were *not* replanned.

## 2 METHODS

### 2.A Adaptive planning process

**Figure 1:**
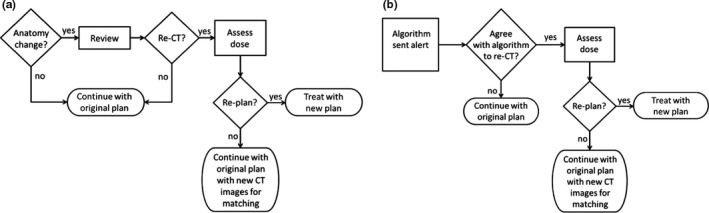
(a) Flowchart of current image review and adaptive planning process used at our institution. (b) The effect of using a computer aid to review images offline.

As part of our institutional guideline, the CBCT match to the planning CT on the first treatment day must be reviewed by the radiation oncologist or the clinical specialist in radiation therapy (CSRT). For the remaining treatment, the radiation therapists are responsible for monitoring anatomy changes that may occur, as mentioned before. Figure 1(a) shows a flow chart of the offline adaptive process at London Regional Cancer Program (LRCP). If external contour differences are 1 cm or more (over approximately one‐quarter of the VMAT 360° arc range) between the current CBCT and the planning CT, a physicist is called to review the image matches in Aria Offline Review (Varian Medical Systems, Palo Alto CA, USA). Image reviews spanning several days are required to assess the trend and duration of anatomical changes. The physicist in collaboration with the CSRT then makes a decision whether or not to re‐CT based on changes in the external contour and/or the target volume, the maximum dose to the spinal cord and other critical organs in the treatment plan and how well the immobilization mask is fitting. If a decision is made to re‐CT the patient, the physicist imports the beams and contours from the original treatment plan onto the new CT scan using Pinnacle Dynamic Planning (Pinnacle v 9.8, Philips, Fitchburg WI, USA). The dose distribution is calculated using the original monitor units on the new CT scan and compared to the original plan. If the dose distribution is substantially hotter as judged by the physicist, or if the dose to critical organs is compromised, the radiation oncologist reviews the dose calculation and a decision is made to replan. If a replan is not warranted, then the new planning CT scan is exported to the treatment unit to use for subsequent image guidance. The purpose of a more automated CBCT comparison tool is to reduce the time and judgment involved in deciding whether the adaptive process is necessary. Figure 1(b) shows the change to the process if an alert was sent to the clinician(s) directly. In this case, the algorithm compares CBCT images quantitatively in the background and tracks changes through software, which suppresses (or skips) the review step in Fig. 1(a). A re‐CT is recommended based on numerical decision criteria, reducing the time taken by staff to review images offline. It would then be a quick check by the radiation oncologist, CSRT or physicist to verify that the algorithm made a reasonable recommendation. The reduction in time by staff will be most relevant for cases where it is obvious that action is *not* required, i.e., cases that should *not* be flagged for review. It should be noted that the CBCT comparison algorithm proposed in this work does not include dose calculation. Therefore, the comparison tool is an anatomical alarm and dose impact assessment would only take place once a re‐CT is ordered.

### 2.B Patient data and CBCT comparison

The imaging guideline at our institution for head and neck cancer treatment is to use daily IGRT: CBCT twice per week (including day 1) and orthogonal kV radiographs on all other days. Daily CBCT is used depending on the case, e.g., proximity of high dose to critical structures such as spinal cord, brain stem, optic structures, and parotid glands. As mentioned previously, we analyzed a total of 30 patients: ten patients for algorithm training and 20 patients for algorithm validation. Ethics approval was obtained for chart review and access to image data sets. Of the 20 patients that were replanned, 13 patients were rescanned because of weight loss, four patients had setup issues, two patients had swelling and one patient had early tumor response. All patients were treated with two 360° VMAT arcs on Varian linear accelerators (21iX or TrueBeam, Varian, Palo Alto CA, USA). The CBCT images were exported from Offline Review and were imported into the gamma comparison software developed in‐house. During treatment, the CBCT is rigidly registered to the planning CT using bony landmarks by the radiation therapists and couch shifts are applied with no action level. Since all CBCT are coregistered to the planning CT in Offline Review, no further image preprocessing steps are required for the gamma comparison. Setting the planning CT scan as the reference scan posed potential problems due to CT number discordance between cone‐beam and helical CT imaging. CBCT numbers are affected by scattering conditions in the patient and are less accurate than those obtained by fan‐beam helical CT. We therefore opted to use the CBCT on fraction 1 as the reference image set to which all subsequent CBCT scans are compared. If there is a replan during treatment, the CBCT on the first day of subsequent treatment is set as the new reference.

As part of treatment plan quality assurance for VMAT, a combination of dose difference and distance‐to‐agreement (DTA), called gamma analysis,^9^ is often used to compare the planned dose distribution to the dose as delivered by the treatment machine.^14^ We repurposed the gamma analysis technique to highlight changes in patient *anatomy* instead of dose, as imaged by CBCT. We use DTA and CT number difference criteria, where CT number contrast is expressed in Hounsfield units (HU). The mathematical formulation of gamma analysis is well‐established.^9^ Briefly, the gamma analysis is a quadratic combination of the distance between the pixels being compared and the difference in their CT‐number value, scaled by DTA and HU‐difference parameters. The main feature of the gamma comparison is that any pixels that have γ>1 correspond to a failure^9^ and the gamma map can show regions of anatomy mismatch visually. Gamma analysis identifies how well two 3‐D data sets match in the context of user‐supplied criteria. These parameters designate how well a pixel must match its immediate surroundings to be considered a *pass*, where as any pixels not meeting these criteria are deemed a *failure*. Although it is a computationally intensive calculation ($O\left(N^{2}\right)$), optimizations to the algorithm,^15^ and adopting graphics processing units (GPUs) can speed up the analysis by several orders of magnitude.^16^ In our lab, commercially available graphics hardware (NVidia GeForce GTX 780) allows gamma computations comparing two CBCT image sets (384 × 384 × 70 voxels) to be completed in less than 5 s.

### 2.C Match quality parameter (MQP)

**Figure 2:**
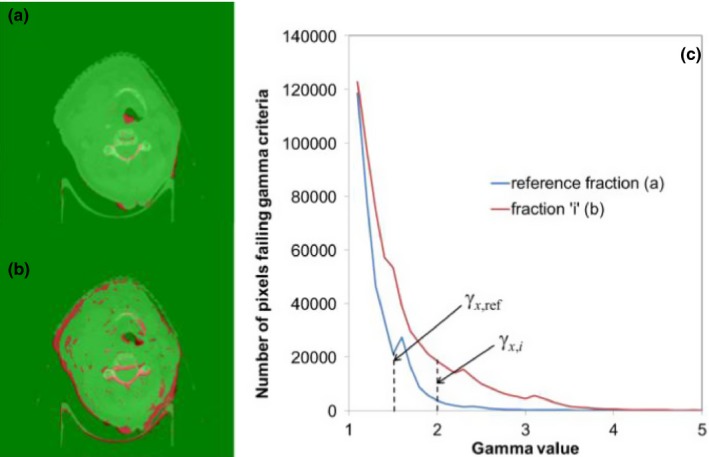
Illustration of gamma (γ) maps and the derivation of match quality parameter. (a) Gamma map from comparing CBCT acquired during first fraction and second fraction where CBCT is acquired. (b) Gamma map from comparing CBCT acquired during the first fraction and some later fraction. (c) Histograms of pixels failing the gamma criteria from both gamma maps. The match quality parameter is defined as the difference between reference percentile gamma value and that from subsequent fractions. Gamma map color scale: γ < 1 green; γ > 1 red.

Instead of calculating a pass rate as is commonly done for dose quality assurance,^14^ we opted to analyze the number of *failed pixels* (γ > 1). Forming a histogram that plots the number of failures by gamma value provides an abundance of measures that can be used to assess how well two image sets match each other, and hence whether it is necessary for a clinician to review the treatment. To be practical, we propose using a single parameter that can be plotted against fraction number to track the quality of the anatomy match throughout the treatment course. Figures 2(a) and 2(b) show gamma maps from one of the patients from the training set using 3 mm DTA and 30 HU, where regions of weight loss are evident in Fig. 2(b). Note that the gamma value is evaluated in 3‐D coordinate space and only one 2‐D slice is shown for simplicity. Figure 2(c) shows the corresponding histograms of γ>1 generated from each map. We calculate the *x* ^th^ percentile of the histograms and then take their difference, namely:

(1) $$MQP_{x,i}=\gamma_{x,\mathrm{ref}}-\gamma_{x,i}$$

where $MQP_{x,i}$ is defined as the match quality parameter for the *i* ^th^ fraction calculated from the *x* ^th^ percentile of the failed‐pixel histogram. The value $\gamma_{x,\mathrm{ref}}$ is the gamma value corresponding to the *x* ^th^ percentile of the histogram from the reference CBCT comparison: i.e., the gamma map generated from the comparison of the CBCT on fraction 1 and the second CBCT. The value $\gamma_{x,i}$ is the gamma value corresponding to the *x* ^th^ percentile of the histogram from the comparison of the CBCT on fraction 1 and some later fraction i. If there is a replan, then $\gamma_{x,\mathrm{ref}}$ is reset to the gamma comparison between the first and second CBCT acquired *after starting the new plan*. As illustrated in Fig. 2(c), anatomy mismatch results in more pixels that fail the gamma criteria, causing the failed‐pixel histogram to shift to the right. Therefore, the reason for the subtraction in eq. (1) is that we are mainly interested in quantifying the *difference* between image comparisons, where a negative MQP shows that the CBCT match during a later fraction is worse than the reference match. In order to ensure that the MQP values are not affected by failed pixels located well outside of the patient anatomy (e.g., due to ring and/or streak artifacts), we use a masked region defined as the external contour from the planning CT structure set plus 1 cm margin. This contour is then limited in the superior/inferior direction by the extension of the Clinical Target Volume (CTV) of the highest dose prescription, which is the treatment region of most concern clinically. Plotting the MQP with fraction number gives a 1‐D display of whether the online anatomy match is deteriorating as treatment progresses. In principle, if there are increased changes in anatomy during treatment, the MQP values will decrease and the plot should show a downward trend. Then, the problem is to use this plot to determine a decision threshold to indicate that a re‐CT may be required.

### 2.D Definition of re‐CT decision criteria

**Figure 3:**
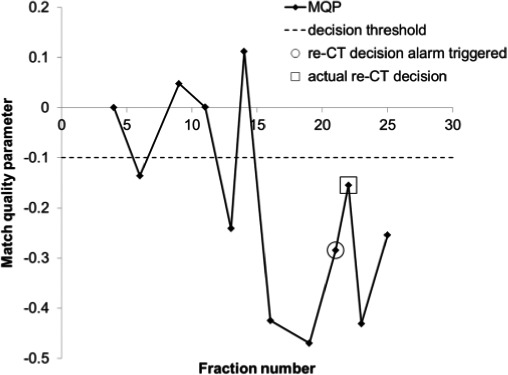
Graph of match quality parameter with fraction number from one of the patients from the training phase. The gamma criteria used was 3 mm DTA and 30 HU along with the 80^th^ percentile from the failed‐pixel histograms.

As a starting point, gamma maps were generated using gamma criteria of 3 mm for DTA and 30 HU for CT number difference for each CBCT; and MQP were calculated from the histograms of γ > 1 using eq. (1) for the ten patients in the training data set. Figure 3 shows a plot of the MQP_80_ with fraction number for one of the patients, meaning that the MQP values were calculated from eq. (1) using the 80^th^ percentile of the failed‐pixel histogram. The MQP oscillates for the first few fractions, and then there is a sudden downward trend that suggests that the online match is beginning to deteriorate systematically from the plan at fraction 16. After fraction 16, the anatomical changes are fairly consistent until fraction 25, which is the last fraction that the original plan was treated before switching to the new plan. We retrieved the date of the actual re‐CT decision order that was entered by the CSRT (or radiation oncologist) from retrospective chart reviews. This date is indicated by the open square at fraction 22 in Fig. 3. Using the re‐CT order date as the benchmark, we defined a decision threshold such that *three consecutive MQP values must be less than or equal to a predetermined threshold*, to trigger a re‐CT recommendation within ±3 fractions of the actual re‐CT order date. The decision threshold is indicated by the dashed line in Fig. 3. The three‐fraction condition is satisfied in Fig. 3 where the decision threshold is chosen to trigger the re‐CT decision at fraction 21 (i.e., within 3 days of fraction 22). From Fig. 3, the lowest threshold that would trigger a re‐CT recommendation would be −0.285, as indicated by the open circle at fraction 21. If the threshold is set lower than this, then the algorithm would not recommend a re‐CT at all for this patient, which is incorrect since this patient was actually replanned. Therefore, in the training phase of the algorithm, we need to find the MQP threshold value that gives the best trade‐off in sensitivity and specificity for all ten patients in the training set. In order to quantify the algorithm's predictive power, we define the following:

1. True Positive (TP): Algorithm triggered a re‐CT recommendation within ±3 fractions of the actual re‐CT order date, provided a re‐CT order could be justified based on review of the CBCT match to the planning CT.
2. True Negative (TN): Algorithm did not trigger a re‐CT recommendation and no re‐CT was ordered. This includes patients that did not need a re‐CT order at any point during treatment as well as patients that were replanned and did not need any further re‐CT after the replan.
3. False Positive (FP): Algorithm triggered a re‐CT recommendation when no re‐CT was ordered (i.e., *false alarm*). This also includes cases when the algorithm triggered a re‐CT recommendation *sooner* than the 3 fraction range as defined in true positive.
4. False Negative (FN): Algorithm did not trigger a re‐CT recommendation where a re‐CT was actually ordered due to changes in anatomy (i.e., a *miss*). This also includes cases when the algorithm triggered a re‐CT recommendation *after* the 3 fraction range as defined in true positive.

The number of TP, TN, FP, and FN were scored for several combinations of DTA and CT number gamma criteria along with the percentile of the failed‐pixel histograms, while varying the MQP decision threshold between −0.001 and −0.5 in steps of −0.001. Note that positive decision thresholds do not make sense for this application since we do not want the algorithm to trigger re‐CT recommendations when it is obviously not necessary. For the gamma criteria, we used 3 mm DTA while varying the HU‐difference criteria. Also, we kept 30 HU‐difference constant while varying the DTA. For each gamma criteria combination, we calculated MQP values for each fraction with varying percentiles of the failed‐pixel histograms (recall Fig. 2(c) and eq. (1)). Receiver‐operator characteristic (ROC) analysis^13^ was then used to determine the optimum parameter combination, namely, gamma criteria, percentile and MQP decision threshold.

## 3 RESULTS

### 3.A MQP plots

**Figure 4:**
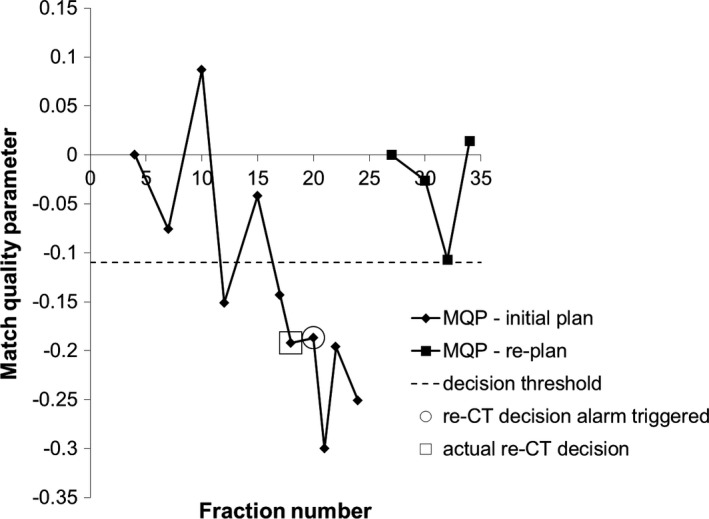
Match quality parameter plot with fraction number for one typical patient in the training data set (different patient from Fig. 3). Gamma criteria 3 mm DTA, 30 HU and 80^th^ percentile from the failed‐pixel histograms were used.

**Figure 5:**
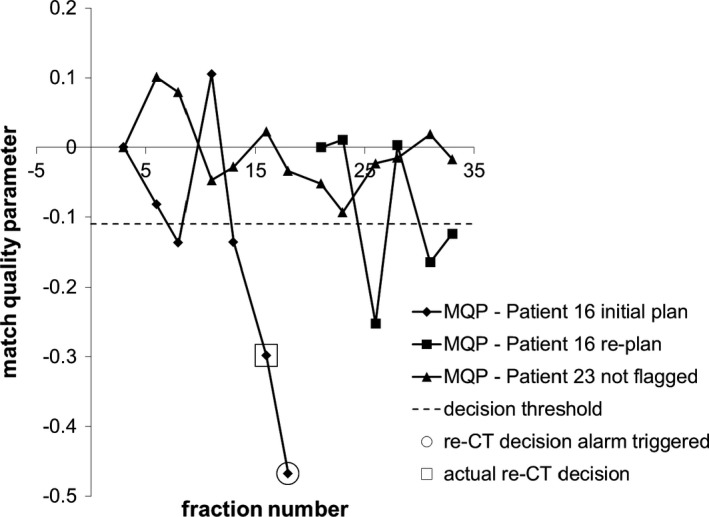
Match quality parameter plot with fraction number for two of the test patients (different from the training patients): One patient that was replanned and one patient not flagged for review during treatment. Gamma criteria 3 mm DTA, 30 HU and 80^th^ percentile from the failed‐pixel histograms were used.

The training data set consisted of ten head and neck cancer patients that were replanned during their treatment course. One patient was replanned twice because of early tumor response. This resulted in 11 possible true positives (TP) in this data set. Two of the patients did not have sufficient CBCT scans after their replan to qualify for the three‐fraction MQP decision condition so these were excluded from the analysis. This resulted in eight possible true negatives (TN). The test patient data set consisted of ten additional patients that were replanned and ten patients that were not replanned or reviewed for possible re‐CT. This resulted in ten possible TP and 16 possible TN since four of the replanned patients did not have sufficient CBCT data after their replan. Figure 4 shows the MQP_80_, i.e., the MQP calculated from the 80^th^ percentile of the failed‐pixel histograms, for one of the patients in the training data set using our initial choice of gamma criteria (3 mm, 30 HU). The MQP plot shows a downward trend when there are changes in anatomy during the original treatment plan. Then, correctly, no re‐CT recommendations were made during the replanned treatment. Note that the MQP resets for the new plan (fraction 27 for this patient). The MQP decision threshold value that gave the best trade‐off in sensitivity and specificity for all ten patients (−0.11) using 3 mm DTA and 30 HU gamma criteria is indicated by the dashed line. The optimum MQP threshold of −0.11 correctly recommended re‐CT in 9 of 11 possible instances. In the patient who had early tumor response, the MQP values were negative but the magnitude of values was not below the threshold and hence the decision condition was not met. This could happen when the anatomy on fraction 1 is substantially different from the planning CT, such that the algorithm did not detect differences from the reference match. In the other patient, retrospective review of the online matches showed that external contour differences were much smaller for this patient; however, a decision was made to re‐CT and replan anyway. Figure 5 shows the MQP_80_ for two of the replanned test cases: one patient that was replanned and one patient that was not flagged for review by the radiation therapists. The same MQP decision threshold value from Fig. 4 is included. Overall, the MQP plots for the patients that were replanned exhibit a similar downward trend as shown in Fig. 4. From the test cases, the decision threshold correctly identified that all patients needed further review, i.e., there were no false negatives. However, the algorithm triggered too early in four instances and triggered a false positive in one of the patients after the replan for a total of five false positives. The MQP plots for the patients that were not replanned fluctuate near zero (indicated by the triangles in Fig. 5), which would be expected, except for two patients. In both of these patients there were external contour differences but they were less than 1 cm in magnitude, which were not flagged for review by the radiation therapists because of our in‐house guideline. This means that the algorithm (correctly) did *not* recommend a re‐CT in eight of ten patients that were not replanned.

### 3.B ROC analysis

**Figure 6:**
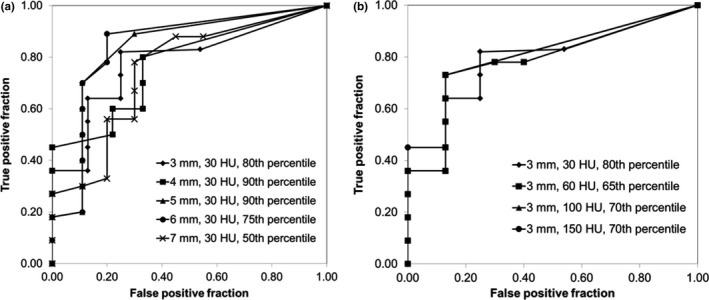
ROC curves for the ten patients in the training phase: (a) 30 HU and varying DTA gamma criteria and (b) 3 mm DTA and varying HU‐difference gamma criteria. The percentile chosen is optimized for each DTA criteria.

**Figure 7:**
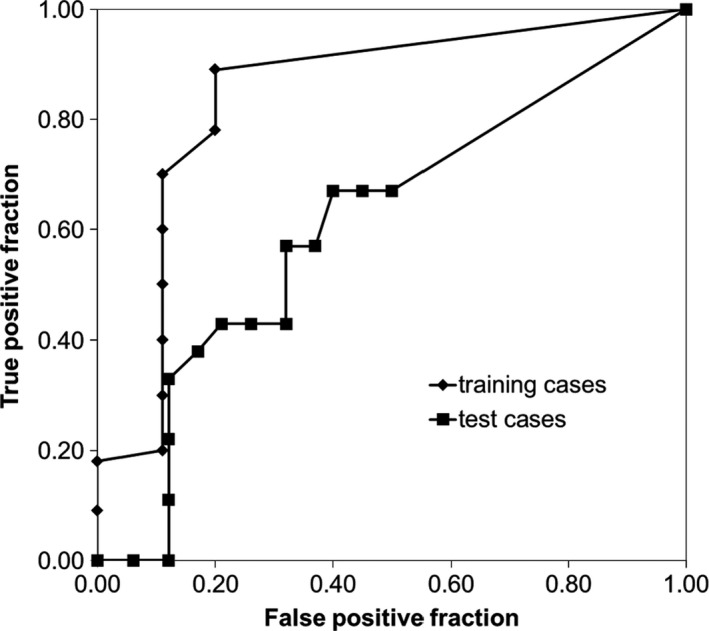
ROC curves for 6 mm DTA and 30 HU gamma criteria for the training patients compared to the test patients.

**Figure 8:**
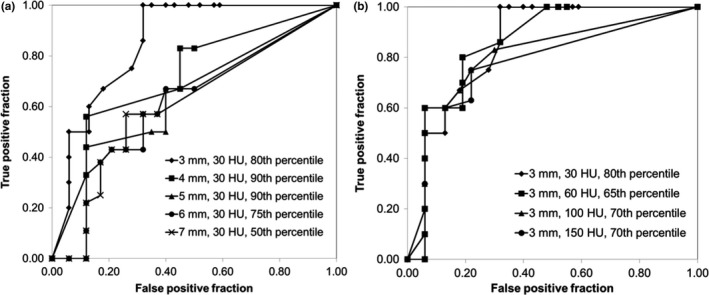
ROC curves for the 20 test patients: (a) 30 HU and varying DTA gamma criteria and (b) 3 mm DTA and varying HU gamma criteria. The percentile from the failed‐pixel histograms for each curve is the same as that shown in Fig. 6.

Figure 6 shows ROC curves for the ten patients used to train the algorithm. For each gamma criteria, the percentile of the failed‐pixel histogram used to calculate the MQP is optimized for sensitivity and specificity. Figure 6(a) shows the effect of varying the DTA while keeping the CT number difference constant at 30 HU, while Fig. 6(b) shows the effect of varying the HU‐difference criteria while keeping the DTA constant at 3 mm. Note that inclusion of the 3 mm DTA and 30 HU criteria on both panels is intentional. In Fig. 6(a), there does not appear to be any decrease in algorithm sensitivity until a DTA of 7 mm is used. In Fig. 6(b), the ROC curves show that the algorithm performance is similar for all HU‐difference criteria, except that the sensitivity is slightly increased for the 30 HU criteria. Of all the ROC curves, the gamma criteria of 6 mm DTA and 30 HU appears to give the best trade‐off in sensitivity and specificity, where a true positive fraction (TPF) of 0.89 and a false positive fraction (FPF) of 0.2 are achievable (note that TPF is the sensitivity and FPF is 1 − specificity). Unfortunately, as shown in Fig. 7, the algorithm performance was suboptimal using the training set criteria (6 mm DTA, 30 HU, 75^th^ percentile of failed‐pixel histogram) when applied to the test patients. The explanation is the increase in the number of false negatives in this set of patients, i.e., the algorithm fails to detect changes in anatomy in this particular set of patients. Therefore, we deduce that 6 mm DTA would not be acceptable in the ability to track changing anatomy in general, if this algorithm is to be used clinically. We applied the remaining parameter sets from Fig. 6 to the test patients and the result is shown in Fig. 8. Clearly from Fig. 8(a), the algorithm performs best when 3 mm DTA and 30 HU gamma criteria are used to recommend re‐CT. In this case, the algorithm (correctly) recommended re‐CT in all ten patients but at the expense of a few false positives, while increasing the DTA gives more false negatives. The ROC curve for the 3 mm DTA and 30 HU gamma criteria shows that a TPF of 1.00 (i.e., 100% sensitivity) and a FPF of 0.32 are achievable in the test patients. In Fig. 8(b), the ROC curve for 3 mm DTA and 60 HU gamma criteria also shows a TPF of 1.00 but at a FPF of 0.48, i.e., at the expense of more false positives. Although the 3 mm DTA and 30 HU gamma criteria was not the best performer in the training patients (from Fig. 6), it still provided a reasonable trade‐off in sensitivity and specificity with a TPF and FPF of 0.82 and 0.25 respectively. Therefore, based on the consistency of very good performance across the training and test patients, we suggest using the 3 mm DTA and 30 HU gamma criteria along with using the 80^th^ percentile gamma value from the failed‐pixel histogram to compute the MQP values to reflect our institution's practice.

## 4 DISCUSSION

We have presented an automated method of comparing anatomy changes during the treatment of head and neck cancer. Our current institutional guideline is that if the immobilization mask does not fit properly, the radiation oncologist is contacted and a replan is ordered immediately. Otherwise, if there are external contour differences of 1 cm or more and the immobilization mask still fits well, the radiation therapist contacts a physicist or the clinical specialist in radiation therapy (CSRT) to review images in Aria Offline Review. The physicist or CSRT may need to review several images to determine the relevant time period and approximate VMAT arc range spanning the anatomical differences, and a recommendation is made to the radiation oncologist to review and order a re‐CT if clinically relevant. In the automated process, we used anatomical gamma analysis to compare two CBCT images mathematically. Then, we calculated the histogram of the number of pixels failing the gamma criteria and introduced the match quality parameter (MQP) as defined in eq. (1). From this, we determined the criteria needed to recommend re‐CT comparable to the timing of decisions made within our department's practice. The ROC analysis showed that we can achieve 82% and 100% sensitivity in the training patients and test patients, respectively, at a false positive rate of ~30% using 3 mm DTA and 30 HU gamma criteria.

Although the algorithm shows potential for more efficient management of head and neck cancer patients that undergo anatomical changes during radiation therapy, there are limitations and some details that need future work. The main limitation is that the anatomy on day 1 may be different from the anatomy in the planning CT. If the anatomy has changed between planning and treatment, the failure histograms might be similar and may result in non‐negative or small negative differences in the MQP as determined using eq. (1). Under these conditions, the re‐CT decision condition might not be met. This occurred in one patient in the training data set and resulted in a false negative for some combinations of the gamma criteria. Typically at our institution, the time between CT simulation and treatment is less than 2 weeks for radical treatment cases. Given this time frame, we do not expect the patient's anatomy to have changed substantially between CT simulation and the first treatment day in the vast majority of cases. Since this did not occur in any of the test patients, this was an issue in only one of the thirty patients used in the study. As mentioned previously, the CBCT match to the planning CT on day 1 must be reviewed. Therefore, in the instance that the anatomy changes between simulation and treatment, the clinician can overrule or by‐pass the algorithm and a re‐CT can be ordered if needed. One possible solution to this problem is to compare the CBCT to the planning CT directly, where some preprocessing steps would be required because of the differences in calibration of CT and CBCT Hounsfield numbers. Investigation of the use of deformable image registration to deform the first CBCT to the planning CT, both in order to assess the CBCT as well as improve subsequent comparisons is another potential area of future work. Another limitation is the inconsistency of CBCT image quality and influence of artifacts since the gamma calculation incorporates CBCT Hounsfield number differences. Severe streak artifacts occurred in one of the patients and gave a false negative for certain gamma criteria (but not 3 mm DTA and 30 HU). Some artifacts can cause a CBCT number difference of ±100 HU,^17^ which will certainly affect the gamma calculation and could trigger an alert. However, this was an issue in only one of the thirty patients, and more patient data and experience is needed to determine the proportion of false positives and false negatives that could be due to these limitations. Future work will include further testing of the algorithm on more retrospective cases and eventually prospective cases. Lastly, our algorithm does not solve the replanning problem directly because we did not assess dosimetric impact. It provides a quick method of determining whether adaptive planning should be considered. In other words, the application of this algorithm within the adaptive process is in two steps [recall Fig. 1(b)]: CBCT comparison would be performed daily, and then re‐CT and dose assessment would only be performed after an alert is sent (barring a false alarm). Dose calculation on CBCT poses potential problems because of the limited superior/inferior scan length as well as potential dose inaccuracy near tissue inhomogeneities.^18^ Correlating the MQP with dose metrics is a possible area of future work.

Clinical implementation of the algorithm would not be difficult and can be adapted to reflect an individual clinic's practice. Once the images are imported into the software, the gamma analysis and MQP calculation takes a few seconds and could be done in the background. The clinician(s) would only be alerted if the MQP re‐CT condition was met. Therefore, using this algorithm should not add much session time at the treatment machine. In the case of false positives, the clinician would have the authority to overrule any automated re‐CT recommendation. However, the radiation therapists doing the online match should remain cognizant of weight loss and other patient changes in case the algorithm fails to detect those changes. In those cases, the radiation therapists can overrule the algorithm and request further review if needed. It should be emphasized that the algorithm is *not* meant to replace the clinician's judgment. The purpose of the algorithm is to assist the clinician by flagging patients with deviations in anatomy that potentially have an adverse effect on target coverage and/or overdosing critical organs in a more automated way, while reducing the number of image reviews where it is obvious that a replan would not be necessary.

## 5 CONCLUSION

We have developed a cost‐effective tool to assess anatomical changes in CBCT images using the gamma comparison method. A parameter called match quality parameter (MQP) was introduced and was calculated using the histogram of pixels that fail the CBCT gamma criteria (γ>1). The MQP plotted with fraction number showed a downward trend if the magnitude of anatomical differences increased as the treatment progressed. We proposed that recommending a re‐CT requires three consecutive MQP values to be less than or equal to a numerical decision threshold value. The decision criteria were derived from comparing the timing of the algorithm to the timing of actual re‐CT decisions that were based on expert judgment within our department. The parameter combination of gamma criteria, area under the histogram (percentile) and MQP threshold that gave the best trade‐off in sensitivity and specificity was determined using ROC analysis.

## CONFLICT OF INTEREST

The authors have no relevant conflict of interest to disclose.
